# May Patients Receiving GLP-1 Agonists Be at Lower Risk of Prostate Cancer Aggressiveness and Progression?

**DOI:** 10.3390/cancers17091576

**Published:** 2025-05-06

**Authors:** Julia Drewa, Katarzyna Lazar-Juszczak, Jan Adamowicz, Kajetan Juszczak

**Affiliations:** 1 Department of Urology and Andrology, Collegium Medicum, Nicolaus Copernicus University, 85-094 Bydgoszcz, Poland; 2 Primary Health Care Clinic of the Ujastek Medical Center, Krakow University of Health Promotion, 31-158 Cracow, Poland

**Keywords:** GLP-1, GIP, prostate cancer, obesity, pathogenesis, agonists

## Abstract

Introduction: GLP-1 receptor agonists are valuable therapeutic agents for managing obesity and type 2 diabetes. The link between prostate cancer and obesity was described. The modulation of incretin hormone-dependent pathways may decrease the prostate cancer aggressiveness and progression. Objectives: The purpose of this study was to review and summarize the literature on the role of GLP-1 agonists in prostate cancer. Material & Methods: We performed a scoping literature review of PubMed from January 2002 to February 2025. Search terms included “glucagon-peptide like 1”, “incretin hormone”, “GLP-1 receptor agonist”, and “prostate cancer”. Secondary search involved reference lists of eligible articles. The key criterion was to identify studies that included GLP-1 receptor, incretin hormones, GLP-1 receptor agonists, and their role in prostate cancer development. Results: 77 publications were selected for inclusion in this review. The studies contained in publications allowed us to summarize the data on the role of GLP-1 receptor and it’s agonists in prostate cancer biology and development. The following review aims to discuss and provide information about the role of incretin hormones in prostate cancer pathogenesis and its clinical implication in patients with prostate cancer. Conclusion: Incretin hormone-dependent pathways play an important role in prostate cancer pathogenesis. Moreover, GLP-1 receptor agonists seems to be a promising therapeutical agents when it comes to finding new therapies in patients with more aggressive and/or advanced stages of prostate cancer.

## Simple Summary

Obesity and periprostatic adipose tissue (via adipokine release) play an important role in prostate cancer development. Moreover, diabetes and metabolic syndrome are involved in prostate cancer. GLP-1 receptor agonists (GLP-1RAs) are valuable therapeutic agents for managing obesity and type 2 diabetes. This literature review provides detailed information on the role of incretin hormone-dependent pathways in prostate cancer development, as well as the potential impact of GLP-1RAs on prostate cancer. GLP-1RAs seem to be promising, novel, therapeutical agents, especially in advanced and metastatic prostate cancer when standard therapies become insufficient. Nevertheless, further research is necessary in this field.

## 1. Introduction

Obesity and diabetes are two of the most common non-infectious disorders [1]. The accumulation of an excessive amount of body fat can cause type 2 diabetes, and the risk of type 2 diabetes increases linearly with an increase in body mass index (BMI) [2]. Glucagon-like peptide 1 (GLP-1), an incretin hormone, has multiple metabolic effects. In particular, GLP-1 reduces blood glucose and body weight [3]. GLP-1 receptor agonists (GLP-1RAs), novel agents that are dual and triple hormonal agonists, have emerged as valuable therapeutic agents for managing obesity and type 2 diabetes [1]. GLP-1 receptor agonists provide better glycemic status and body weight control. Also, GLP-1 receptor agonists prevent cardiovascular disorders and improve kidney functioning [4]. Obesity positively correlates with the risk of prostate cancer and its more aggressive forms [5]. Sung et al. [6] found that overweight and obesity is a risk factor for prostate cancer. Obesity causes about 4%–8% of all cancers [7]. Moreover, there is a close relationship between body mass index and the risk of cancer, as well as cancer mortality. For example, the results of Ramadani et al. [8] showed that there was an increased risk of prostate cancer and mortality in individuals with BMI above 25 kg/m^2^. Prostate cancer is the second-most commonly diagnosed cancer [9]. In the case of the European population, prostate cancer remains the most commonly diagnosed cancer (excluding skin cancers) [10]. The results of Wang et al. [11] showed that GLP-1 receptor agonist treatment is associated with decreased risks of several cancers (e.g., prostate, lung, and colon cancer), though these agonists increase the risk of thyroid cancer. A meta-analysis performed by Cui et al. [12] showed that antihyperglycemic drugs such as thiazolidinediones and GLP-1 receptor agonists show benefits in individuals with prostate cancer.

Moreover, GLP-1 receptor agonists diminish the risk of prostate cancer development in comparison with sulfonylureas [13].

The main purpose of the review was to discuss the importance of incretin hormones and GLP-1 receptors in the pathogenesis of prostate cancer. Additionally, the current state of knowledge regarding the effect of GLP-1 receptor agonists on prostate cancer and the potential clinical implications of this therapy in patients with prostate cancer is discussed.

## 2. Incretin Hormones

Incretins are protein hormones that are secreted after nutrient intake and stimulate insulin secretion together with hyperglycemia. There are two incretin hormones: glucagon-like peptide (GLP-1) and glucose-dependent insulinotropic polypeptide (GIP) [14]. The enteroendocrine L cells of the small intestine continuously secrete GLP-1. The GLP-1 receptor (GLP-1R) is a class B G protein–coupled receptor (GPCR) that mediates the action of GLP-1. It predominantly localizes to the cellular membrane of diverse cell types throughout the human body [15]. The enzymatic activity of dipeptidyl peptidase IV (DPP-4) leads to GLP-1 degradation and inactivation. The duration of action of GLP-1 is 1–3 min [16]. GLP-1 receptor activity is mediated via G protein (Gαs), which stimulates adenylate cyclase, leading to increased intracellular level of cyclic AMP (cAMP). cAMP activates a protein kinase called phosphokinase A (PKA), which activates a number of intracellular pathways. Moreover, cAMP activates a process called EPAC (exchange protein directly activated by cAMP). EPAC is important in several cellular processes (e.g., cell adhesion and junction formation, etc.). All the abovementioned intracellular pathways provide insulin release and genetic alteration through GLP-1 [17,18]. Moreover, GLP-1 receptor-dependent pathways mediate intracellular signaling through Gαs.

Signal transduction from the receptor to the cell nucleus depends on ERK1/2 activity. Due to phosphorylation of intracellular proteins, ERK1/2 influences several processes: (1) cell proliferation, (2) cell differentiation, and (3) apoptosis. The cytoplasmatic regulatory pathways are related to β-arrestin activation. On the other hand, the transient translocation to the nucleus is due to PKA activation [18]. GLP-1 stimulates calcium-dependent insulin secretion. This secretion is enhanced by increased Ca^2+^ influx due to several PKA- and EPAC-dependent mechanisms: (1) the inhibition of ATP-regulated K^+^ ion channels, (2) an increase in activity of L-type voltage-gated Ca^2+^ channels (VGCCs), and (3) triggering the opening of non-specific cation channels [18,19,20,21].

GLP-1 receptors (GLP-1Rs) are present in islet β cells and the central nervous system [1].

GLP-1 exerts direct or indirect effects on many organs. On pancreas, stomach, heart, and brain, GLP-1 acts directly, while indirect influence is exerted on muscle and liver [22]. GLP-1 prolongs gastric emptying, enabling a significant decrease in post-meal glycemic excursions. Additionally, GLP-1 suppresses appetite (via GLP-1 receptors that are expressed in multiple regions of the central nervous system). In the pancreas, it increases insulin biosynthesis and β-cell proliferation, as well as suppressing β-cell apoptosis. Due to increased insulin secretion and reduced glucagon secretion, GLP-1 indirectly affects glucose production in the liver (decrement) and also increases insulin sensitivity in muscle (by increasing microvascular recruitment in skeletal muscle, which may potentiate local insulin action) [1,22]. Changes in blood glucose levels lead to the release of GLP-1 into the portal circulation. As a result, there is increased secretion of insulin due to the efferent activity of the vagus nerve. Additionally, insulin secretion is directly activated by incretin hormones (GLP-1 and GIP) via islet β-cell receptors [23]. GLP-1 has a central anorexigenic effect via the reduction of appetite and energy intake, as well as increased satiety [24]. Calanna et al. [25] revealed that the effect of GLP-1 receptor agonists on weight may contribute to an increase in insulin sensitivity and better control of blood glucose.

Administration of GLP-1 receptor antagonists directly into the brain increases food consumption in satiated rats. Moreover, decreased proglucagon gene expression (which is crucial in GLP-1 encoding) leads to heightened appetite and consequential weight gain [26]. GLP-1 reduces gut inflammation via its receptor on intraepithelial lymphocytes, whereas GIP suppresses macrophage-dependent inflammation. Also, adipose tissue is affected by incretin hormones. White adipose tissue is indirectly affected by GLP-1 and GIP. On the other hand, within brown adipose tissue, inflammatory processes and amnio acid and fat metabolism are directly regulated by GIP [27].

## 3. Incretin Hormone Receptor Agonists

GIP and GLP-1 are not used in therapy because of their very short half-life of about 1 to 3 min. This rapid degradation is due to the rapid hydrolysis of these substances by dipeptidyl peptidase 4 (DPP-4). The agonists of GLP-1 receptors are resistant to enzymatic degradation by DPP-4 [28,29]. Several incretin hormone receptor agonists have been introduced into clinical practice for diabetes type 2, as well as for obesity or overweight with at least one weight-related condition (dyslipidemia, hypertension, sleep apnea, diabetes, cardiovascular disease, etc.) treatment. GLP-1 receptor agonists have been divided into three groups depending on their mechanism of action on incretin hormone receptors: (1) via GLP-1 receptors (single-action agonists—liraglutide and semaglutide), (2) via GLP-1 and GIP receptors (dual-action agonist—tirzepatide), and (3) via GLP-1–GIP–glucagon (triple-action agonist—retatrutide). Exendin 4 shows great structural (53% conformity in amnio acid sequence) and functional similarity to human GLP-1. For clinical practice, several GLP-1 receptor agonists have been approved, such as (1) albiglutide, dulaglutide, liraglutide, and semaglutide (GLP-1 analogues) and (2) exenatide and lixisenatide (exendin 4 analogues). Also, a dual GIP–GLP-1 receptor agonist (tirzepatide) has FDA approval [30]. Depending on the duration of action (short- or long-acting), GLP-1 receptor agonists have different efficacy and tolerance. It is crucial to develop GLP-1 agonists that are resistant to DPP-4 and have delayed renal clearance. The abovementioned features can be achieved by appropriate modification of the structure of a given agonist. This allows for a longer half-life and increased GLP-1 receptor-stimulating properties. So far, two GLP-1 receptor agonists showing DPP-4 resistance have been developed (albiglutide and dulaglutide) [30]. A more intense hyperglycemic response is observed during simultaneous activation of GIP and GLP-1 receptors. Frías et al. [31] showed that dual GLP-1 receptor agonists are more effective in proper glycemic control compared to single-action agents. Similarly, dual-action agents better control metabolic homeostasis and appetite [22].

Compared to patients treated with antidiabetic drugs (excluding GLP-1 receptor agonists), patients treated only with GLP-1 receptor agonists have been observed to be at increased risk of certain comorbidities, such as (1) cardiovascular disorders (hypotension, syncope), (2) gastrointestinal disorders and pancreatitis, (3) degenerative joint diseases, and (4) nephrolithiasis and interstitial nephritis.

GLP-1 receptor agonist therapy is associated with a decreased risk of neurocognitive disorders (e.g., dementia, and Alzheimer’s disease), psychotic diseases, and seizures. In addition, a reduced risk of conditions such as cardiovascular diseases, coagulation disorders, and respiratory abnormalities was observed during GLP-1 receptor agonist treatment [32]. Therefore, potential side effects must be carefully considered in patients qualified for such treatment.

It is worth noting that the number of overweight or obese individuals (without coexisting diabetes) treated with GLP-1 receptor agonists is constantly increasing. In the United States population, an increase of over 700% was recorded between 2019 and 2023 [33].

Recently, there has been increasing use of GLP-1 receptor agonists in the treatment of overweight and obesity, as well as in patients with diabetes. The clinical efficacy of this therapy is indisputable, especially in terms of the metabolic profile. On the other hand, the potential effect on carcinogenesis remains unclear. Levy et al. [34] examined how these medications affect cancer risk in people with obesity in a population-based study of over 1.1 million patients. The results revealed that GLP-1 receptor agonists (particularly semaglutide) afforded a significant risk reduction encompassing 25 different cancer types. GLP-1RA use was associated with a protective effect on malignant neoplasms of digestive organs at 5-year follow-up. This effect was particularly pronounced for colorectal and liver/biliary cancers. Additionally, the risk of female and male genital organ malignancies, breast cancer, melanoma, thyroid and endocrine gland cancers, and others (eye, brain, and central nervous system cancers) was reduced with GLP-1 receptor agonists therapy [34].

## 4. Prostate Cancer, Obesity, and Diabetes

The positive association between prostate cancer and obesity has been described in previous meta-analyses. There is also a positive association between the risk of prostate cancer and an increased level of insulin growth factor 1 [28].

### 4.1. Prostate Cancer and Obesity

The strict relationship between obesity and the risk of prostate cancer remains unclear. Prostate cancer is positively correlated with metabolic syndrome and obesity [35]. The more aggressive forms of prostate cancer are more frequent in obese men [35,36]. Moreover, previous clinical studies revealed that obese men are at higher risk of dying of prostate cancer [37]. Obese patients are predisposed to extensive amounts of visceral fat tissue. The prostate gland is covered and surrounded by adipose tissue in an area of approximately 48%. This tissue is called periprostatic adipocyte tissue (PPAT) [38]. The adipose tissue is not considered a passive tissue, but one that exhibits constant activity influencing many pathophysiological processes via paracrine and endocrine signaling (through adipokines). Moreover, adipose tissue has an influence on prostate cancer development and progression [39,40]. Several potential pathomechanisms seem to be critical in prostate cancer pathogenesis in obese individuals: (1) chronic inflammation within adipose tissue surrounding the prostate gland, which intensifies local and systemic inflammatory response, (2) hyperinsulinemia due to excess adipose tissue and obesity, and (3) increased levels of adipokines as a consequence of increased PPAT [41]. The functional prostate gland microenvironment, which is composed mostly of white adipose tissue surrounding prostate (PPAT), takes an active part in physiological conditions and in the development of prostate cancer. PPAT remains an active endocrine tissue, influencing the activity of prostate cells. Additionally, PPAT provides bidirectional modulation of microenvironment activity. PPAT due to adipokine release promotes prostate cancer cell migration and invasion. Additionally, PPAT affects the prostate cancer cells’ aggressiveness and potency [41,42]. Moreover, it is worth noting that in the case of obesity, a constant proinflammatory state is observed. In individuals with normal body weight, the proper balance between pro- and anti-inflammatory processes is maintained. Maintaining the proper balance is possible due to the increased release of anti-inflammatory factors from adipocytes and the extracellular matrix [43]. A predominance of proinflammatory activity is typical in case of weight gain, and is associated with inflammation processes and insulin resistance. This sequel is due to overproduction of adipokines that enhance the proinflammatory response. Also, in obesity, hypertrophic adipocyte cells tend to die, and in consequence the existing inflammation is intensified by released intercellular substances [41,44]. It is worth noting that obesity remains an independent predictive factor of prostate cancer. Also, further progression in advanced stages of prostate cancer is associated with obesity [45].

### 4.2. Prostate Cancer and Diabetes

In individuals with diabetes, the incidence of prostate cancer is decreased. This is most likely due to low serum levels of IGF-1 caused by long-lasting hypoinsulinemia [46]. Lee et al. [47] found prostate cancer-specific mortality and all-cause mortality increased by 29% and 37%, respectively. Also, metabolic syndrome predisposes an individual to a higher risk of prostate cancer [48]. It is worth mentioning that hyperinsulinemia always accompanies metabolic syndrome. Increased levels of serum insulin stimulate epithelial cell proliferation within the prostate gland and enhance cancer migration and invasiveness [49]. Additionally, individuals with metabolic syndrome were characterized by shorter overall survival and shorter time to the transformation to castration-resistant prostate cancer (CRPC) [50].

It is postulated that GLP-1 receptors are involved in inflammation processes, insulin resistance development, and carcinogenesis. Among other things, insulin resistance is based on chronic inflammation mediated by a number of cytokines, such as TNF-β, interleukin 1β, and interleukin 6. A GLP-1 agonist (exendin 4) not only attenuated macrophage infiltration but also inhibited the macrophage secretion of inflammatory cytokines, including TNF-β, interleukin 1β, and interleukin 6 [51]. IL-1 and IL-6 are elevated in obese patients [40]. Moreover, these cytokines remain a risk factor of several cancers (e.g., colorectal and lung cancer) [52]. Activated macrophages present in the tumor microenvironment release IL-1, in parallel with the secretion of adipose tissue [53]. In prostate cancer pathogenesis, inflammation induces remodeling of the extracellular matrix and triggers epithelial-to-mesenchymal transition [54].

Prostate cancer progression and neuroendocrine differentiation is affected by cytokines with proinflammatory features [55]. Moreover, it is well established that tumor development and progression is promoted by inflammatory processes. A previous study showed that IL-6 is associated with cancer development due to inflammatory process modulation [40]. However, IL-6 presents anti-inflammatory properties. An increased level of IL-6 negatively correlates with prostate cancer survival and response to chemotherapy in subjects with castration-resistant prostate cancer (CRPC) or untreated metastatic prostate cancer. In prostate cancer, the increased cell proliferation and suppressed apoptosis is a response to IL-6 [53]. An aggressive prostate cancer phenotype and metastasis development are linked to IL-6 through the regulation of epithelial–mesenchymal transition (EMT) and homing of prostate cancer cells to the bone [56]. Prostate cancer growth is diminished by metformin and GLP-1 receptor agonists (e.g., exendin 4). This reduced tumor growth is due to the suppression of prostate cancer cell proliferation. Moreover, metformin inhibits cancer cell proliferation via apoptosis induction [50]. Moreover, combined therapy (exendin 4 with metformin) is more effective in suppression of prostate cancer development compared to monotherapy of the abovementioned agents. Also, thiazolidinediones and GLP-1 receptor agonists reduce the risk of prostate cancer. Contrastingly, sodium–glucose cotransporter 2 (SGLT2) inhibitors show no correlation with prostate cancer risk [12].

## 5. Prostate Cancer and Incretin Hormones—Dependent Pathway

The incretin hormones present several metabolic impacts (e.g., reduction in weight and glucose level). Moreover, several studies showed that patients with diabetes and cardiovascular disease or at high cardiovascular risk who are treated with GLP-1 receptor agonists had a reduced risk of adverse cardiovascular events [57,58]. In addition to these metabolic effects, incretin hormones appear to influence tumor biology. Human prostate cancer tissue expresses large amounts of GLP-1 receptors [59]. Primary human prostate cancer expresses GLP-1 receptors in in vitro conditions. The results of Stein et al. [59] showed a positive expression of GLP-1 receptors in advanced stages of prostate cancer in humans. Moreover, based on data from the Human Protein Atlas, RNA expression is different in human prostate carcinoma samples: a majority are negative for GLP-1 receptors, and of the common human prostate cancer cell lines, only 22Rv1 and LNCaP cells express GLP-1 receptors at the RNA level [60]. Previous studies on cell lines showed that GLP-1 receptor agonists inhibit signaling pathways engaged in tumorigenesis of prostate cancer through the suppression of proliferation and increase apoptosis of human prostate cancer cells [28,61]. Li et al. [62] showed that exenatide and liraglutide significantly inhibited the proliferation of androgen-sensitive human prostate (LNCaP) cell lines and induced cell apoptosis. Moreover, GLP-1 receptor agonists increased the level of apoptosis-related proteins (Bax and Bcl-2). Moreover, in LNCaP cell lines, the activation of p38 but not ERK1/2 or Akt was observed. These findings show that GLP-1 receptor agonists attenuate prostate cancer growth via regulating the P38 pathway.

**Figure 1:**
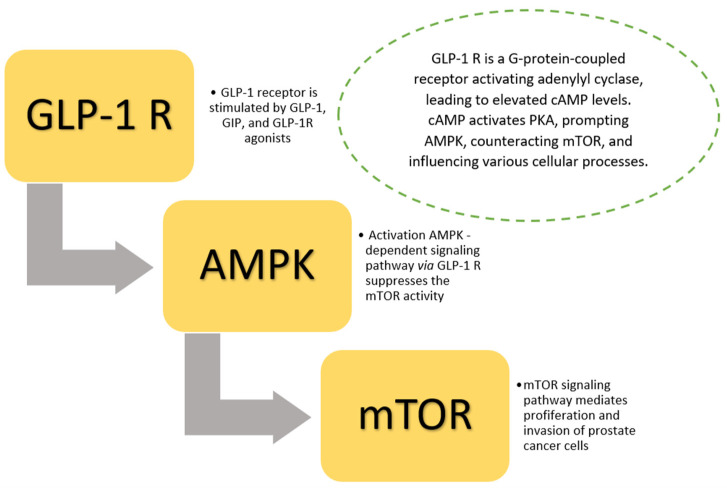
Role of GLP-1 receptors in prostate cancer carcinogenesis (anti-tumorigenic effect) via mTOR-dependent signaling pathway. GLP-1 receptors are expressed in human prostate tissues and cell lines. GLP-1 receptor agonists increase AMPK phosphorylation, leading to a decrement in the levels of p-mTOR 9. This promotes an anti-tumorigenic effect through suppression of cancer cell proliferation and invasion. Abbreviations: GLP-1—glucagon-like peptide 1, R—receptor, GIP—glucose-dependent insulinotropic polypeptide, AMPK—5′AMP-activated protein kinase, mTOR—mechanistic target of rapamycin, PKA—phosphokinase A, cAMP—cyclic adenosine monophosphate.

Similarly, antihyperglycemic drugs (e.g., metformin) and exendin 4 (GLP-1 receptor agonist) inhibit cell proliferation, providing suppression of tumor growth. Nevertheless, these agents present no impact on cell migration [63]. Additionally, human prostate cancer tissue samples and prostate cancer cell lines present positive expression of GLP-1 receptors. The diminished cancer cell proliferation is due to suppression of the ERK-MAPK-dependent pathway by exendin 4 in in vitro and in vivo prostate cancer models [64]. There is a close relationship between obesity and the pathogenesis of prostate cancer. Moreover, obesity is associated with chronic inflammation. Chronic inflammation promotes the development of several types of solid cancers and might contribute to prostate carcinogenesis [65]. It is worth noting that GLP-1 receptor agonists suppress the systemic inflammation response, which may be implicated in the pathogenesis of prostate cancer. A nationwide cohort study performed by Skriver et al. [61] revealed that GLP-1 receptor agonist use was inversely associated with prostate cancer risk. The authors indicated that GLP-1 receptor agonists may present protective effects against prostate cancer. In carcinogenesis, the mTOR-dependent signaling pathway plays a crucial role in proliferation, migration, and invasion. The results of Shigeoka et al. [66] showed that GLP-1 receptor expression levels were significantly inversely associated with the Gleason score of human prostate cancer tissues. GLP-1 receptor agonists increase AMPK phosphorylation, leading to decrements in the levels of p-mTOR 9, cyclin B, and p34 [67]. This promotes anti-tumorigenic effects via suppression of cancer cell proliferation and invasion (Figure 1). Moreover, in vivo and in vitro studies showed that the GLP-1 receptor analogue exendin 4 enhanced prostate cancer response to ionizing radiation (IR) via induction of G2–M cycle arrest in prostate cancer cells in a dose-dependent manner [68] (Figure 1).

In response to nonsteroidal antiandrogen therapy (enzalutamide), the PI3K/Akt/mTOR-dependent pathway is activated. Additionally the PI3K/Akt/mTOR pathway is associated with resistance to enzalutamide therapy [69]. Moreover, the GLP-1 analogue exendin 4 inhibits the growth of prostate cancer cells through suppressing PI3K/Akt/mTOR signaling pathway. The results of Wenjing et al. [69] showed that exendin 4 with enzalutamide suppressed prostate cancer cell growth in comparison with monotherapy based on enzalutamide. Enzalutamide triggers Akt and mTOR levels. GLP-1 receptor agonists (in combined therapy with enzalutamide) diminish the levels of Akt and mTOR, leading to decrements in nuclear androgen receptor localization. Contrary, no impact of GLP-1 receptor analogue monotherapy on nuclear androgen receptor was observed [69].

In metastatic prostate cancer, docetaxel-based chemotherapy, which stabilizes microtubules, remains a first-line treatment. The results of Eftekhari et al.’s [68] in vitro experiments on the androgen-sensitive human prostate cancer cell line LNCaP revealed a synergistic effect of docetaxel and liraglutide based on induction of cell cycle arrest and in consequence apoptosis. Moreover, the cells that were exposed to the combination of docetaxel and liraglutide presented diminished intracellular phosphorylation of signaling proteins [11].

**Figure 2:**
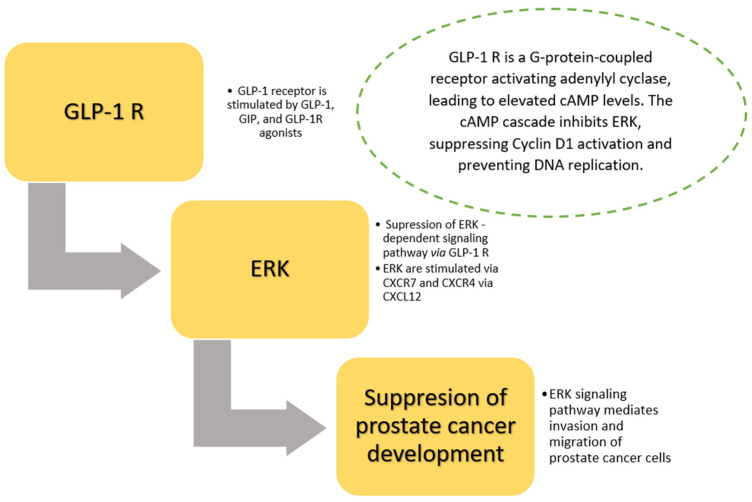
Role of GLP-1 receptors in prostate cancer carcinogenesis via ERK-dependent signaling pathway. Prostate cancer cells have positive expression of GLP-1 receptors. Several signaling pathways via GLP-1 receptors (e.g., ERK-MAPK and PI3K/AKT/mTOR) regulate cell proliferation and apoptosis (see also Figure 1). CXCR7 promotes the activation of ERK1/2, stimulating the process of invasion and migration of cancer cells. Abbreviations: GLP-1—glucagon-like peptide 1, R—receptor, GIP—glucose-dependent insulinotropic polypeptide, ERK—extracellular signal-regulated kinase, CXCL—ligand of chemokine receptor, CXCR—chemokine receptor CXCR, cAMP—cyclic adenosine monophosphate.

When discussing the role of GLP-1 receptor agonists in the regulation of pathways influencing prostate cancer carcinogenesis, it is worth mentioning dipeptidyl peptidase 4 (DPP-4), which also regulates the activity of the ERK1/2- and mTOR-dependent pathway. The inactivation of GLP-1 and GIP by DPP-4 in consequence leads to diminished binding of ligands of the chemokine receptor CXCL-12 to chemokine receptors CXCR4 and CXCR7 [28]. CXCR7 promotes the activation of ERK1/2, stimulating the process of invasion and migration of cancer cells (Figure 2). On the other hand, the process of proliferation and invasion of cancer cells is enhanced by the activation of the mTOR-dependent pathway. mTOR is activated via Akt (protein kinase B). Akt is activated simultaneously through 1) CXCR7 (direct stimulation) and 2) indirectly via CXCR4 and phosphoinositide 3-kinase (PI3K) [28] (Figure 2).

In the development of cancer, the process of neo-angiogenesis is very important. GLP-1 shows pro-angiogenic activity. The results of Aronis et al.’s [70] study on human umbilical vein endothelial cells (HUVECs) showed that GLP-1 promotes angiogenesis in a dose-dependent manner. Moreover, the inhibition of Akt, PKC, or src significantly decreases GLP-1-induced angiogenesis.

## 6. Prostate Cancer and GLP-1 Receptor Agonists

Glucagon-like peptide 1 receptor agonist therapy is associated with lower risks of several cancers (e.g., prostate, lung, and colon cancer) and a higher risk of thyroid cancer [11]. GLP-1 agonists exhibit multifaceted effects on prostate cancer development through several pathways. GLP-1 receptor agonists increase cAMP levels via activation of GLP-1 receptor and adenylyl cyclase. The cAMP-dependent cascade activates several pathways, such as (1) inhibition of ERK, suppression of cyclin D1 activation, and prevention of DNA replication, and (2) simultaneously cAMP activates PKA, prompting AMPK, counteracting mTOR, and influencing various cellular processes [70].

In prostate cancer, due to the anti-proliferative feature of P27, the cell cycle is suppressed. Moreover, P27 modulates the RhoA-dependent signaling pathway and in consequence alters proper cell migration. In response to GLP-1 receptor agonists, the increased level of intracellular cAMP inhibits the proliferative cascade and further cell invasion [71,72,73]. P27 inhibits mTOR. Therefore, GLP-1 receptor agonists possess the ability to suppress cell proliferation and tissue invasion by increasing the intracellular level of cAMP [71]. The results of Cui et al. [12] showed that antidiabetic drug (thiazolidinediones or GLP-1 receptor agonists) administration may have benefits in prostate cancer based on randomized clinical trials. Liraglutide, a GLP-1 receptor agonist, reduces the risk of prostate cancer in comparison with patients assigned to placebo in addition to standard care (LEADER study) [14,49].

It is worth noting that GLP-1 receptor agonists show interesting and promising complementary effects to standard treatment methods used in patients with prostate cancer, The GLP-1 receptor agonist may intensify and strengthen the effect of irradiation, hormonotherapy, or chemotherapy. The results of He et al. [68] showed positive expression of GLP-1 receptors in prostate and cell lines. In in vitro and in vivo studies, irradiation suppresses proliferation (via cell cycle arrest), and this effect is additionally promoted by exendin 4. The strength of the effect depends on the dose of the exendin 4.

Similarly to the positive effects in irradiated individuals, the GLP-1 receptor agonist exendin 4 diminishes resistance to the novel antiandrogen enzalutamide. This observation suggests that GLP-1 receptor agonists may be used in patients with advanced forms of prostate cancer when a reduced response to enzalutamide therapy is observed [69]. In patients with prostate cancer during chemotherapy, GLP-1 receptor agonists seem to be useful. The results of Eftekhari et al. [68] revealed that combined therapy based on docetaxel and liraglutide (GLP-1 receptor agonist) may enhance the chemotherapy response in metastatic prostate cancer (use of a lower dose with maintained efficacy and reduced toxicity). The standard first-line chemotherapy in prostate cancer individuals is docetaxel [74]. The multidirectional action of GLP-1 agonists and promising results of experimental studies indicate that GLP-1 agonists enhance the response to docetaxel.

This promising effect of GLP-1 agonists during chemotherapy suggests that these drugs may be helpful in the treatment of metastatic castration-resistant prostate cancer. GLP-1 receptor agonists appear to allow for a reduction in the dose of docetaxel while maintaining clinical efficacy and reducing toxicity during chemotherapy in patients with metastatic prostate cancer.

In patients with castration-resistant prostate cancer, enzalutamide (an androgen receptor inhibitor) improves overall survival. Additionally, in patients with metastatic hormone-sensitive prostate cancer (mHSPC), combined hormonotherapy based on standard hormonal drugs and novel antiandrogens improves survival, resulting in an increase in the percentage of participants alive at 5 years from 57% to 67% [75]. Additionally, in patients with metastatic castrate-resistant prostate cancer and progression following docetaxel chemotherapy, further life-prolonging treatment options should be taken into account in selected cases (e.g., novel anti-androgens, radium-223, and olaparib, etc.).

Dihydrotestosterone (DHT) presents an insulinotropic effect in mouse and human islets. DHT activates GLP-1 receptors and DHT intensifies the incretin effect of GLP-1. The extracellular androgen receptors, which are activated by DHT in β cells, enhance glucose-stimulated insulin secretion (GSIS) by increasing islet cAMP and activating protein kinase A [76]. There are some functional relationships between androgen receptors and the GLP-1 receptor-dependent pathway. Androgens amplify the incretin effects of GLP-1 through activation of the GLP-1 receptor [77].

GLP-1 receptor agonists affect the regulatory pathways mediated through androgen receptors. Additionally, GLP-1 agonists present the ability to sensitize to docetaxel-based chemotherapy. These facts indicate a high predictive value of this group of drugs in the treatment of patients with obesity and prostate cancer.

## 7. Future Perspectives

In obese patient with prostate cancer, the risk of cancer progression is more potent due to chronic inflammation, which is strictly related to increased secretion of proinflammatory adipokines and overproduction of reactive oxygen species. Periprostatic adipose tissue, which is part of the surrounding microenvironment for prostate cancer, participates in bidirectional interplay with cancer cells via the paracrine route [38]. Considering the fact that activation of GLP-1 receptor-dependent pathways demonstrates anti-inflammatory effects and reduces the degree of insulin resistance (as well as the amount of free oxygen radicals produced), it seems that GLP-1 receptor agonists will find wide application in the treatment of obesity, especially in patients with prostate cancer. An interesting and potential mechanism of action of GLP-1 receptor agonists on prostate cancer seems to result from a direct effect on obesity and metabolic status of the patient with prostate cancer. In a patient with obesity, the reduced risk of prostate cancer development and/or progression may be due to reduced secretion of adipokines from periprostatic adipose tissue in response to treatment with GLP-1 receptor agonists. Similarly, the reduced degree of aggressiveness of existing prostate cancer during GLP-1 receptor agonist treatment is possible. Additionally, these drugs are a promising direction of treatment, taking into account the fact of synergistic action with hormonal therapy (e.g., new antiandrogen agents—enzalutamide), chemotherapy (e.g., docetaxel), and irradiation. Despite the promising results of experimental studies to date, it is still necessary to conduct randomized clinical trials to assess the clinical effects of GLP-1 receptor agonists in patients with prostate cancer.

## 8. Conclusions

Current data highlight the fundamental role of obesity, periprostatic adipose tissue, and metabolic syndrome in prostate cancer development and progression. Thus, GLP-1 receptor agonists seem to be an interesting and promising avenue with potential therapeutic benefits in patients with prostate cancer.

## List of Abbreviations

Akt	protein kinase B
AMPK	5′AMP-activated protein kinase
BAX	BCL2-associated X protein (apoptosis regulator)
BMI	body mass index
cAMP	cyclic adenosine monophosphate
CRPC	castration-resistant prostate cancer
CXCL	ligand of chemokine receptor
CXCR	chemokine receptor CXCR
DDP-4	dipeptidyl peptidase 4
DHT	dihydrotestosterone
EPAC	exchange protein directly activated by cAMP
ERK	extracellular signal-regulated kinase
FDA	Food and Drug Administration
GIP	glucose-dependent insulinotropic polypeptide
GLP-1	glucagon-like peptide 1
GLP-1 RAs	glucagon-like peptide 1 receptor agonists
GPCR	G protein–coupled receptor
GSIS	glucose-stimulated insulin secretion
HRR	homologous recombination repair
HUVECs	human umbilical vein endothelial cells
IL-1	interleukin 1
IL-6	interleukin 6
IR	irradiation
JAK	Janus tyrosine kinase family
LNCaP	androgen-sensitive human prostate cells
MAPK	mitogen-activated protein kinase
mHSPC	metastatic hormone sensitive prostate cancer
mTOR	mechanistic target of rapamycin
PI3K	phosphoinositide 3-kinase
PKC	protein kinase C
PPAT	periprostatic adipose tissue
SGLT2	sodium–glucose cotransporter 2
SKP2	S-phase kinase-associated protein 2
Src	Src family of protein tyrosine kinases
STAT	signal transducer and activator of transcription
TNF-β	tumor necrosis factor β
VGCCs	voltage-gated calcium channels
